# *Phytophthora capsici* Epidemic Dispersion on Commercial Pepper Fields in Aguascalientes, Mexico

**DOI:** 10.1100/2012/341764

**Published:** 2012-04-26

**Authors:** Adrián Zapata-Vázquez, Mario Sánchez-Sánchez, Alicia del-Río-Robledo, Héctor Silos-Espino, Catarino Perales-Segovia, Silvia Flores-Benítez, Mario Martín González-Chavira, Luis Lorenzo Valera-Montero

**Affiliations:** ^1^Instituto Tecnológico El Llano Aguascalientes, Ags., Km 18, Carr. Ags.-S.L.P., 20330, Mexico; ^2^CIRCE-INIFAP, Km 6.5 Carr. Celaya-San Miguel de Allende, 38110 Celaya, Gto., Mexico

## Abstract

Chili pepper blight observed on pepper farms from north Aguascalientes was monitored for the presence of *Phytophthora capsici* during 2008–2010. Initially, ELISA tests were directed to plant samples from greenhouses and rustic nurseries, showing an 86% of positive samples. Later, samples of wilted plants from the farms during the first survey were tested with ELISA. The subsequent survey on soil samples included mycelia isolation and PCR amplification of a 560 bp fragment of ITS-specific DNA sequence of *P. capsici*. Data was analyzed according to four geographical areas defined by coordinates to ease the dispersal assessment. In general, one-third of the samples from surveyed fields contained *P. capsici*, inferring that this may be the pathogen responsible of the observed wilt. Nevertheless, only five sites from a total of 92 were consistently negative to *P. capsici*. The presence of this pathogen was detected through ELISA and confirmed through PCR. The other two-thirds of the negative samples may be attributable to *Fusarium* and *Rhizoctonia*, both isolated instead of *Phytophthora* in these areas. Due to these striking results, this information would be of interest for local plant protection committees and farmers to avoid further dispersal of pathogens to new lands.

## 1. Introduction

Chili pepper blight due to *Phytophthora capsici* and *Verticillium* spp. [1] has been present in commercial pepper fields in Aguascalientes and neighboring states in Mexico [2]. Nevertheless, there is a lack of detailed data on the degree of incidence related to spatial distribution from Mexican areas where this *Phytophthora* is prevalent. Some approaches have been developed for Aguascalientes [3], Zacatecas [2], and Chihuahua [4, 5]. From these studies only one [4] deals with some indicators of incidence of *Phytophthora capsici* according to one region. Since pepper blight is so devastating for pepper farms, it is worthy to study its incidence through a survey [6]. Therefore, the purpose of this work is to add more information on the spatial distribution of this pathogen on the northern part of Aguascalientes and describe its actual incidence on chili pepper farms.

## 2. Materials and Methods

### 2.1. Plant and Soil Sampling

Whole plants showing severe wilting, collapsed still-green leaves, and root rot were collected from commercial fields. All of the plants collected in the field showed flowers and fruits. Additionally, 36 plantlet samples from two greenhouses and 16 rustic nurseries were included due to suspicious contamination only for ELISA testing (not for fungi isolations). Collection of plant samples in the field was done five times (once each month) on the same fields (92 geographical positions) from April to September 2008. Some data was missing for the first time of sampling (26 sites) due to late planting. Data was clustered into four groups according to geographic closeness and access roads. During 2009, soil samples from 82 sites within chilli pepper fields were taken with a post hole digger from 0–30 and 30–60 cm deep diggings, rending a total of 164 samples. Both plant and soil samples were taken from northern Aguascalientes (Cosío, Tepezalá, Rincón de Romos and Pabellón de Arteaga) within the parallels 22° 00′ 47′′ and 22° 23′ 25′′ North and 102° 14′ 57′′ and 102° 19′ 11′′ West, where most chilli pepper fields were found. Coordinates for geopositioning the fields of the collected plant and soil samples were recorded.

### 2.2. ELISA Detection of *Phytophthora* on Wilted Chili Plants

Immunological detection through ELISA was performed as described by the supplier of the reagent kit for *Phytophthora* detection in plants (CAB 92600/0500 and ACC00948 from AGDIA). Stems of plants showing root rot (wilted) and controls with normal appearance were sliced with a knife and ground with a mortar. After grinding, 100 *μ*L of sap was placed in Eppendorf tubes plus 1 mL of the extraction buffer GEB2 from the kit and mixed gently. At the same time, mycelia from *Phytophthora capsici* cultures were used as positive control together with the positive control included in the kit. A scrap from cultures about 1 cm^2^ and 500 *μ*L GEB2 were placed inside an Eppendorf tube and ground with a crystal bar. After grinding, tubes were left in vertical position for 10 min to allow particles to sink and reach the bottom. The rest of the protocol was followed according to the directions of the supplier.

### 2.3. Fungal Isolates from Wilted Chili Plants

Fungi isolates were obtained from crown segments of wilted plants with rot roots and lesions on the stem that retained leaves and fruits. Plants were taken fresh from commercial fields (22 during 2010) and washed using tap water and commercial soap. Segments (5–10 mm) from the stem having visible lesions were cut with a razor blade and disinfected with 1% sodium hypochlorite (5–10 min) and rinsed with sterile distilled water for 1-2 min; then, the segments were dried on sterile paper napkins. In order to check for the microorganisms present in the plants, samples were placed on V8-agar (200 mL V8 + 800 mL distilled water + 0.2 g CaCO_3_ + 20 g agar + 4 g sucrose) for 4–7 days in the darkness. In order to obtain *Phytophthora*, selective medium PARP (V8-agar + 5 mg Pimaricin + 250 mg Ampicillin sodium + 10 mg Rifamycin sodium + 50 mg PCNB) [7–9].

### 2.4. Fungal Isolates from Soil

**Media Used**

Acidified Potato Dextrose Agar (PDA + 16 g agar + 250 *μ*L lactic acid) and a PARP variant (V8-agar + 5mg Pimaricin + 250 mg Ampicillin sodium + 10 mg Rifamycin sodium + 30 mg Cefalexin) were fixed. In order to count fungi CFUs, one gram of soil was dissolved on sterile water (10 mL final volume) and vortexed. Once the soil particles descended by gravity, a volume of the supernatant was taken and diluted 1/10. From that dilution 1 mL was spread over the acidified PDA and left for 48 h at room temperature. Colonies were randomly chosen by marking dots with permanent marker, placing a 25-point grid (as a guide) on the bottom of the Petri dish. Microscopic view (4x objective lens) was centered on each dot for colony quantification. Fungi genera were determined as previously described [10] using a 40x objective lens.

*Phytophthora capsici *was isolated using autoclaved fresh peanuts as trap. First, 16 g of soil samples plus 10 mL sterile water was placed in Petri dishes. Then, peanut halves were placed in the Petri dishes together with the soil samples and incubated at room temperature during 48 h. After incubation, peanut halves were placed on the PARP variant for at least 48 h, and once a culture was identified as *Phytophthora*, monosporal or hyphal tip cultures were transferred to a fresh PARP medium, as many times as required, until isolated. Half-an-inch agar dishes containing mycelia from the isolates were cultivated on acidified PDA Petri dishes covered with *≈*6 g ground peanut dried at 36°C plus 6 mL sterile water with antibiotics (30 mg/L Cefalexin + 30 mg/L Rifampicin + 5 mg/L Pimaricin) for 24 h.

### 2.5. PCR Test of Isolates from Soil

DNA from soil isolates was extracted as described elsewere [11]. Briefly, mycelia desiccated at 36°C for 24 h were ground on a mortar with liquid nitrogen. Then, 100 mg of ground mycelia plus 800 *μ*L of extraction buffer (0.2 M Tris, 0.05 M EDTA pH 7.5, 0.2 M NaCl, 2% CTAB and 60 mL 5% Sarcosyl) was placed into a 1.5 mL Eppendorf tube. Tubes were incubated at 65°C during 15 min under continuous gently shaking. After that, 600 *μ*L phenol : chloroform : iso-amyl alcohol (24 : 25 : 1) was added to the tubes and incubated at −20°C for 10 min. Tubes were centrifugated at 13,000 g during 15 min. The supernatant was placed in a fresh tube in order to repeat the previous step. Next, 600 *μ*L of 70% ethanol was added and incubated overnight at −20°C. After that, tubes were centrifugated at 13,000 g during 15 min and the supernatant was discarded. Pellets were washed with 70% ethanol and dried at room temperature. DNA was resuspended in 40 *μ*L TE.

PCR was performed as described by [12]. The primers used amplify a 560 bp fragment from specific *Phytophthora capsici* ITS sequences (PC-1: 5′-GTCTTGTACCCTATCATGGCG-3′ and PC-2: 5′-CGCCACAGCAGGAAAAGCATT-3′). Reaction mixture included 12.5 *μ*L 2x GoTaq Green Master Mix (Promega 2800, Woods Hollow Road Madison WI 53711 USA), 2.5 *μ*L from both primers (10 *μ*M PC-1 and PC-2), 3 *μ*L of genomic DNA, and 7 *μ*L of nuclease-free water (Promega) for a 25 *μ*L final volume. Temperatures of the thermocycler were set as follows: preheat at 94°C during 4 min; 35 cycles including 30 sec at 94°C for denaturing, 30 sec at 70°C for annealing, and 1.5 min at 72°C for polymerization; a final extension at 72°C for 7 minutes. Once the PCR was concluded, samples were run in 1.5% agarose SB gel, with 240 V during one hour.

### 2.6. Statistical Analysis

ELISA results from greenhouse and nurseries were combined and analyzed using chi-square for an equal chance of a positive/negative response (*χ* ^2^, 1 df, *e*_1_ and *e*_2_ = 50%). ELISA results from four geographical zones were analyzed as a contingency table. In both cases, the observed significance level (*P*) was recorded.

## 3. Results

### 3.1. ELISA Detection of *Phytophthora* on Wilted Chili Plants

Plantlets from greenhouse and nursery: initially, 36 samples were selected from plantlets showing either wilting or lesions from two greenhouses (10 samples from cultivars: Caloro, Hungaro, Guajillo, Pasilla, and Puya), and 16 rustic nurseries from poor farmers (26 samples from the same cultivars but Caloro) were tested for the presence of the pathogen. Only one sample out of six from the first greenhouse was positive to the presence of *Phytophthora* while the four samples from the second greenhouse were all positives. Furthermore, all of the samples from nurseries were positive to the test. This was a possible indication that these 86% positive samples may be the starting point for soil contamination of the Aguascalientes chilli fields. Since previous field trips (in 2007) have shown that there was no field free of wilted plants with root rot (5–90%) and information of *Phytophthora capsici* dissemination in chilli fields of Aguascalientes is rather scarce, the expected values of positives were at least 50% from either the nurseries or the greenhouses (data combined as G + N in Table 1).

Plants from the fields: data from the five dates of sampling was combined within each cluster or zone. Zones are described from north to south. In the first zone, located within 22° 21′ 27′′ and 22° 23′ 19′′ North and 102° 15′ 56′′ and 102° 17′ 46′′ West, 36% of the samples were positive. In the second zone, placed within 22° 20′ 43′′ and 22° 21′ 12′′ North and 102° 14′ 57′′ and 102° 16′ 58′′ West, 39.3% were positive. In the third zone, placed within parallels 22° 18′ 26′′ and 22° 18′ 56′′ North and 102° 15′ 40′′ and 102° 16′ 06′′ West, 32.7% were positive. Finally, in the fourth zone located within 22° 18′ 26′′ and 22° 18′ 57′′ North and 102° 15′ 40′′ and 102° 16′ 06′′ West 51.6% were positive. Zones were analyzed as contingency table not including either greenhouse or nursery data (Table 1). Zone 4 had the highest percentage of positives to *Phytophthora*. Nevertheless, this data does not reflect the damage observed on the fields because they were not surveyed for the number of wilted plants or production of commercial fruits. In some cases, the damage was so intense that the whole field was plowed before the crop cycle was completed. Taking together the results of the four zones, only five out of 92 geographical sites consistently did not show the presence of the pathogen on the successive sampling. This is an alarming result, since most of the farmers move to new fields that were not used for chilli pepper, leaving contaminated patches with the pathogen in the northern Aguascalientes.

### 3.2. Fungal Isolates from Wilted Chili Plants

Plants for fungi isolation during 2010 were collected from the same geographical area (176 plants from 22 different fields) as the previous two years; the criteria for previous plant sampling prevailed. Since this type of wilting is due to the attack of fungal complexes, *Phytophthora* was not the only genera isolated. Actually, its counts appear to be lower, probably because the others are aggressive at the time of colonizing the media. *Fusarium* and *Rhizoctonia* have their own contributions for the wilting and root rot as previously described [1]. The rest may be saprophytes or contaminants of the rotten areas (Table 2).

The results of isolation not necessarily reflect all of the events of the process from infection to death of chilli plants due to *Phytophthora*. González-Chavira [13] initially tried to establish a relationship in Guanajuato (Mexico) of this kind but the number of *Phytophthora* isolates was rather low (2%) as compared to *Fusarium spp. *(65%) and *Rhizoctonia solani *(33%). Later, this author moved to PCR for detection of *Phytophthora* from fresh plants [14]. Some of the same pathogens were found as a complex on plants showing similar symptoms (brown-black discolored collar with root rots causing permanent wilting and plant death) in Europe. A three-year survey on pepper showing wilting and discoloration (755 plants from 120 farms, Spain) showed that *Phytophthora capsici *(18%), *Sclerotium rolfsii *(83%), *Rhizoctonia solani*, *Verticillium dahliae *(33%), and other potential pathogens were present [6].

### 3.3. Fungal Isolates from Soil and PCR Confirmation of *P. capsici*

**Figure 1:**
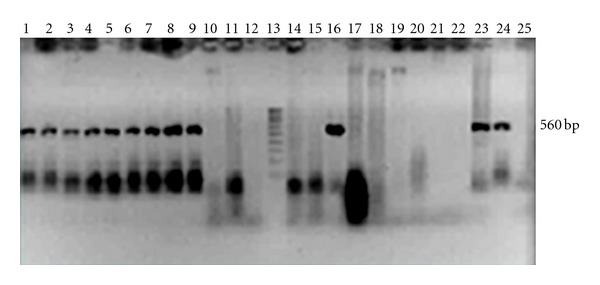
Positive detection of *Phytophthora capsici* from soil samples through PCR amplification of a specific 560 bp ITS fragment (lanes 1–7). Lane 13: molecular weight marker, lanes 24 and 25: positive and negative control, respectively. Lanes 8, 9, 16, and 23 are duplicates from the same samples.

Initial positive counts on acidic PDA of *Phytophthora capsici* were 28% (46 out of 164 soil samples), *Fusarium* sp. 45.7% (75/164), and *Rhizoctonia* sp. 17.7% (29/164). Other potential pathogens (*Verticillium*,* Pythium, *and* Rhizopus*) were not recorded due to their low number. Specific trapping with peanut and *Phytophthora capsici* isolation on PARP, allowed confirming its presence through PCR (Figure 1; Table 3). In this case, it was difficult to obtain positives without trapping *Phytophthora*, since only 1.8% was evident from 164 samples. Trapping with peanut helped in the detection, since 28% of a subsample (25 soil samples) was positive.

## 4. Conclusions

Plantlets positive to *Phytophthora* taken either from greenhouse or nursery were recorded as 86% (31/36); this figure indicates that they may be the primary source for the local spreading of the pathogen in pepper fields of Aguascalientes.

ELISA test showed that one-third of pepper blight samples from the field were positive to the presence of *P. capsici*. Nevertheless, most of the farms showed at least one positive during successive sampling of the same geographical site. Exemptions to the rule were five farms, corresponding to five geographical sites from a total of 92. Therefore, most farms from north Aguascalientes during 2008–2010 were detected as contaminated with *Phytophthora capsici*, distributed approximately the same on three out of four geographical zones. Only zone 4 showed about 50% of ELISA positives. Confirmation on the presence of *Phytophthora* through pathogen isolation and PCR supports ELISA data, although they were done one year later.

Wilting of the two-thirds not positive samples to *Phytophthora* may be attributable to fungus *Fusarium* and *Rhizoctonia* isolated alone or as a complex in this work. Other work includes to *Verticillium* and *Pythium* in the complex [1]. Since our surveys covered most of the pepper farms on the north Aguascalientes and all of the surveyed farms had wilted spots with similar symptoms (wilting with hanging still-green leaves and root rot), attention and preventive procedures should be devoted to avoid spreading of more than one pathogen associated to the blight to new farms. Additionally, detailed data on local dynamics of the disease are necessary [5, 15] to support the work of the local committee for plant protection CESAVEA.

## Figures and Tables

**Table 1:** Results from ELISA test for *Phytophthora capsici* on chili pepper plant samples from greenhouses, nurseries, and farms located in four geographical zones in the northern area of Aguascalientes, Mexico.

ELISA	Greenhouse	Nursery	G + N	Zone 1	Zone 2	Zone 3	Zone 4
Positive	5	26	31	54	22	18	98
Negative	5	0	5	96	34	37	92

% (+)	50	100	86.1	36.0	39.3	32.7	51.6

*χ* ^2^ =			18.78	11.38			
*P *=			1.47E−05	0.0098			

**Table 2:** Fungi and yeast genera identified on isolates from lesions of wilted plants with root rot of chilli pepper collected in the northern area of Aguascalientes, Mexico.

Genera	Counts	Percentage
*Phytophthora*	7	1.76
*Fusarium*	42	10.58
*Rhizopus*	128	32.24
*Alternaria*	36	9.07
*Aspergillus*	53	13.35
*Penicillium*	85	21.41
*Candida*	20	5.04
*Rhizoctonia*	26	6.55
Total	397	100

**Table 3:** Counts of *Phytophthora capsici* cultures from soil samples taken in four geographical zones in the northern area of Aguascalientes, Mexico.

Media	Zone 1	Zone 2	Zone 3	Zone 4	(+)	Total**	%
Acidic PDA*	29	3	10	2	44	164	27
PARP-V8*	4	0	2	1	7	25	28
PARP-PCR confirm	2	1	0	0	3	164	1.8
Peanut-PCR confirm	3	1	2	1	7	25	28

*Counts in Petri dish.

**Total soil samples or dishes.
